# Absolute and Functional Iron Deficiency in the US, 2017-2020

**DOI:** 10.1001/jamanetworkopen.2024.33126

**Published:** 2024-09-24

**Authors:** Yahya M. K. Tawfik, Hayley Billingsley, Ankeet S. Bhatt, Iman Aboelsaad, Omar S. Al-Khezi, Pamela L. Lutsey, Leo F. Buckley

**Affiliations:** 1 Department of Pharmacy Services, Brigham and Women’s Hospital, Boston, Massachusetts; 2 Department of Clinical Pharmacy, College of Pharmacy, King Saud University, Riyadh, Saudi Arabia; 3 Division of Cardiovascular Medicine Department of Internal Medicine, University of Michigan, Ann Arbor; 4 Division of Clinical Research, Kaiser Permanente, Oakland, California; 5 Department of Clinical Research, El-Maamourah Chest Hospital, Alexandria, Egypt; 6 Department of Pharmacy Practice, College of Pharmacy, Qassim University, Qassim, Saudi Arabia; 7 Division of Epidemiology & Community Health, School of Public Health, University of Minnesota, Minneapolis

## Abstract

**Importance:**

The prevalence of absolute and functional iron deficiency among adults in the US is unknown.

**Objective:**

To estimate the prevalence of absolute and iron deficiency and iron supplement use in the US across age, sex, and comorbidity categories.

**Design, Setting, and Participants:**

This cross-sectional study analyzed data from the National Health and Nutritional Examination Survey (NHANES) 2017 to 2020 prepandemic cycle. Participants included noninstitutionalized, civilian women and men aged 18 years or older who had available serum ferritin, iron, and unsaturated iron binding capacity measurements. Data analysis was performed from March 21, 2023, to July 5, 2024.

**Exposure:**

Absolute iron deficiency and functional iron deficiency.

**Main Outcomes and Measures:**

Absolute iron deficiency was defined as serum ferritin less than 30 ng/mL regardless of transferrin saturation. Functional iron deficiency was defined as serum ferritin greater than or equal to 30 ng/mL with transferrin saturation less than 20%. The prevalence of absolute and functional iron deficiency was estimated among all adults in the US and separately among women and men according to age category (>18 years to <50 years, 50-65 years, and ≥65 years) using recommended sample weights and sampling design factors to provide estimates representative of the national, noninstitutionalized civilian population. The 95% CIs were calculated using the Korn-Graubard method.

**Results:**

A total of 8021 US adults (mean age, 48 years; 95% CI, 47-49 years; 52% [95% CI, 50%-53%] female) were included in this analysis. An estimated 14% (95% CI, 13%-15%) of adults in the US met the criteria for absolute iron deficiency, and an estimated 15% (95% CI, 14%-17%) met the criteria for functional iron deficiency. Among US adults without anemia, heart failure, chronic kidney disease, or current pregnancy, the estimated prevalence of absolute iron deficiency was 11% (95% CI, 10%-11%), and that of functional iron deficiency was 15% (95% CI, 14%-17%). The prevalence of functional iron deficiency exceeded that of absolute iron deficiency in all US adults except women younger than 50 years. Iron supplement use ranged from 22% (95% CI, 12%-37%) to 35% (95% CI, 29%-42%) of women with iron deficiency and 12% (95% CI, 5%-21%) to 18% (95% CI, 8%-32%) of men with iron deficiency depending on age.

**Conclusions and Relevance:**

These findings suggest that absolute and functional iron deficiency affect a large proportion of American adults even in the absence of anemia, heart failure, or chronic kidney disease. Further research on the role of functional iron deficiency in adverse health outcomes and on iron deficiency screening strategies is needed.

## Key Points

**Question:**

What is the prevalence of absolute and functional iron deficiency among adults in the US?

**Findings:**

This cross-sectional, nationally representative survey of 8021 US adults estimated that absolute iron deficiency affects 14% and functional iron deficiency affects 15% of adults in the US. Absolute iron deficiency most often affects young women, whereas functional iron deficiency affects women and men of all ages.

**Meaning:**

These findings suggest that absolute and functional iron deficiency represent common public health problems, and further research on the role of functional iron deficiency in adverse health outcomes and on iron deficiency screening strategies is needed.

## Teaser

This cross-sectional study evaluates the prevalence of absolute and iron deficiency and iron supplement use in the US across age, sex, and comorbidity categories.

## Introduction

Iron plays an important role in many biological functions.^1,2^ Absolute iron deficiency results from a severe reduction or absence of iron stores. Functional iron deficiency occurs in the presence of adequate iron stores but insufficient iron availability (thus, it is not a true deficiency of body stores). Although most research has focused on adverse outcomes among people with iron deficiency and anemia, iron deficiency affects nonerythropoietic tissue, such as skeletal and cardiac muscle, in the absence of anemia.^1,2^ Iron deficiency has been associated with restless leg syndrome, decreased physical capacity, impaired neurocognitive function, heart failure, all-cause mortality, and other adverse outcomes independent of anemia.^1^

Infants and children, people of childbearing potential, and people with certain conditions, such as anemia, chronic kidney disease, and heart failure, have high rates of iron deficiency.^3,4,5,6^ The prevalence of iron deficiency outside these groups and the potential underlying causes remain unclear. Moreover, the prevalence of functional iron deficiency has not been determined. Iron deficiency can be prevented or treated through dietary interventions or nonprescription iron supplements, but the current use of nonprescription iron supplements and dietary iron intake and iron deficiency prevalence have not been studied. These estimates are needed to identify populations at risk of iron deficiency–related adverse outcomes, inform screening recommendations, and establish research priorities. We estimated the prevalence of absolute and functional iron deficiency and iron supplement use in the US from 2017 to 2020 across age, sex, and comorbidity categories.

## Methods

### Study Population

The study population included participants of the nationally representative National Health and Nutrition Examination Survey (NHANES) 2017 to 2020 prepandemic cycle who were older than 18 years and had available serum ferritin, iron, and unsaturated iron binding capacity measurements. The 2017 to 2020 prepandemic cycle includes pooled data from the 2017 to 2018 and 2019 to 2020 cycles because the 2019 to 2020 cycle field operations were halted after completion of data collection in 18 of 30 locations owing to the COVID-19 pandemic. The 2017 to 2020 prepandemic cycle comprises a nationally representative sample of the civilian, noninstitutionalized population in the US over a 3.2-year period. A full report describing the methods for the combined 2017 to 2018 and 2019 to 2020 cycle data has been published previously.^7^

The National Center for Health Statistics institutional review board approved the NHANES study. All participants provided written informed consent. All data can be obtained from the NHANES website.^8^ The present cross-sectional analysis was approved by the Mass General Brigham Institutional Review Board. This study follows the Strengthening the Reporting of Observational Studies in Epidemiology (STROBE) reporting guidelines.

### Iron Deficiency Definitions

Serum ferritin levels, serum iron levels, and unsaturated iron binding capacity were measured using an electrochemiluminescence immunoassay.^9,10,11^ Transferrin saturation was calculated as serum iron levels divided by total iron binding capacity (sum of serum iron and unsaturated iron binding capacity) multiplied by 100.^11^ Absolute iron deficiency was defined as serum ferritin level less than 30 ng/mL (to convert to micrograms per liter, multiply by 1).^1,12^ Functional iron deficiency was defined as transferrin saturation less than 20% with serum ferritin level greater than or equal to 30 ng/mL.^1,12^ An alternative serum ferritin threshold of less than 15 ng/mL for absolute iron deficiency was used in sensitivity analyses.

### Other Variable Definitions

Race and ethnicity were self-reported by participants by selecting 1 of the following categories specified in NHANES: Asian American, Mexican American, non-Hispanic Black, non-Hispanic White, or other (ie, any other race or ethnicity not otherwise specified, including multiracial). Race and ethnicity were included in this study to identify disparities in iron deficiency prevalence and treatment. Cigarette use was self-reported. Anemia was defined as hemoglobin concentration of less than 12.0 g/dL (to convert to grams per liter, multiply by 10) for women and less than 13.0 g/dL for men.^13^ Heart failure history was self-reported. Estimated glomerular filtration rate (eGFR) was calculated using the age-based and sex-based 2021 Chronic Kidney Disease Epidemiology Collaboration equation, and chronic kidney disease was defined as an eGFR of less than 60 mL/min/1.73 m^2^. Pregnancy status was self-reported or, for those who denied current pregnancy or were unsure, defined using a urine pregnancy test. Iron supplement use was reported by participants and, when possible, verified against pill bottles during the interview. Any product that contained iron was considered an iron supplement.

Food security was categorized as full, high, marginal, low, and very low using responses to the US Food Security Survey Module questionnaire.^14,15^ Alcohol intake status was categorized as nondrinkers (participants responding no to ever had a drink of any kind of alcohol), low-to-moderate drinkers (<2 drinks per day for men and <1 drink per day for women), and heavy drinkers (≥2 drinks per day for men and ≥1 drink per day for women). Nutrients and food components were calculated from each food or beverage using the 2017 to 2018 and 2019 to 2020 USDA Food and Nutrient Database for Dietary Studies. Dietary iron intake was calculated as the mean of values obtained at two 24-hour dietary recall interviews separated by 3 to 10 days.^16^ Participants’ responses were included in the analysis only if they were deemed reliable in both the in-person and telephone assessments.^17,18^

### Statistical Analysis

Data analysis was performed from March 21, 2023, to July 5, 2024. We estimated the prevalence of absolute and functional iron deficiency among all adults in the US and separately among women and men according to age category (>18 years to <50 years, 50-65 years, and ≥65 years) using recommended sample weights and sampling design factors to provide estimates representative of the national, noninstitutionalized civilian population.^7^ The 95% CIs were calculated using the Korn-Graubard method as recommended by the National Center for Health Statistics’ Data Presentation Standards for Proportions.^19^ Any 95% CI estimates that did not meet National Center for Health Statistics standards are presented and flagged as such, with statistical significance defined as *P* < .05. We repeated sensitivity analyses using a ferritin threshold of less than 15 ng/mL to define absolute iron deficiency instead of less than 30 ng/mL. We estimated the prevalence of anemia, heart failure, and eGFR less than 60 mL/min/1.73 m^2^ in the overall adult population and according to iron deficiency status (replete, absolute deficiency, or functional deficiency) using similar sample weights and design factors.

We assessed factors related to the risk of absolute iron deficiency or functional iron deficiency vs iron replete status using multinomial regression models that accounted for the sampling and design of NHANES. All models included age category (<50 years or ≥50 years); sex; the interaction between age category and sex; self-reported race and ethnicity category; alcohol use; body mass index; presence of anemia, heart failure, eGFR less than 60 mL/min/1.73 m^2^, or pregnancy; dietary iron intake (log_2_-transformed); iron supplement use; and food security status. We evaluated those same factors for associations with serum ferritin levels (log_2_-transformed) and transferrin saturation levels using multivariable linear regression models. We also evaluated factors associated with iron supplement use and dietary iron intake. These models included the same factors as previous models except that iron supplement use or dietary iron intake were excluded and iron deficiency status was added. Stata statistical software version 17.0 (StataCorp) was used for all analyses.

## Results

### Participant Characteristics

The study sample included 8021 adult participants of the 2017 to 2020 NHANES prepandemic cycle, which represented an estimated 244.6 million US individuals (eFigure in Supplement 1). The mean age of the sample was 48 years (95% CI, 47-49 years), 21% (95% CI, 18%-23%) were aged 65 years or older, and 52% (95% CI, 50%-53%) were women (Table 1). Approximately 27% of participants reported marginal, low, or very low food security (Table 1). Prevalent anemia (7%; 95% CI, 6%-8%), heart failure (3%; 95% CI, 2%-3%), and eGFR less than 60 mL/min/1.73 m^2^ (6%; 95% CI, 5%-7%) were uncommon in the overall adult population.

**Table 1.** Characteristics of Included Adult Participants in the National Health and Nutritional Examination Survey 2017-2020 Prepandemic Cycle

Characteristic	Participants, % (95% CI)		
	Overall (N = 8021)	Women (n = 4147)	Men (n = 3874)
Age, mean (95% CI), y	48 (47-49)	49 (47-50)	47 (46-49)
Age categories			
>18 to <50 y	53 (50-56)	52 (49-55)	54 (51-57)
≥50 to <65 y	26 (25-28)	26 (25-28)	27 (24-29)
≥ 65 y	21 (18-23)	22 (20-24)	19 (17-22)
Sex			
Women	52 (50-53)	NA	NA
Men	48 (47-50)	NA	NA
Race and ethnicity			
Asian American	6 (4-8)	6 (4-8)	5 (4-7)
Mexican American	9 (6-12)	8 (6-11)	9 (7-12)
Non-Hispanic Black	11 (8-14)	12 (9-15)	10 (8-13)
Non-Hispanic White	63 (58-68)	63 (58-68)	63 (58-68)
Other Hispanic	8 (6-9)	8 (6-10)	8 (6-10)
Any other race or ethnicity not otherwise specified, including multiracial	4 (3-5)	4 (3-5)	5 (4-5)
Food security status			
Full	73 (70-75)	71 (69-74)	74 (71-79)
Marginal	11 (9-13)	11 (10-13)	10 (8-13)
Low	9 (8-10)	10 (9-11)	8 (7-9)
Very low	7 (6-9)	8 (7-9)	7 (6-9)
Current cigarette smoking	17 (15-19)	15 (13-17)	18 (16-21)
Atherosclerotic cardiovascular disease	8 (7-10)	7 (6-9)	10 (8-12)
Hypertension	54 (51-56)	52 (49-55)	56 (53-59)
Heart failure	3 (2-3)	2 (2-3)	3 (2-4)
Type 2 diabetes	11 (10-12)	10 (9-11)	13 (12-15)
Body mass index, mean (95% CI)^a^	30 (29-30)	30 (30-30)	30 (29-30)
eGFR, mean (95% CI), mL/min/1.73 m^2^	97 (95-98)	97 (95-98)	96 (95-97)
eGFR <60 mL/min/1.73 m^2^	6 (5-7)	6 (5-9)	5 (4-6)

Abbreviations: eGFR, estimated glomerular filtration rate; NA, not applicable.

^a^ Body mass index is calculated as weight in kilograms divided by height in meters squared.

### Prevalence of Iron Deficiency Among Adults in the US

An estimated 14% (95% CI, 13%-15%) of US adults had absolute iron deficiency, and an estimated 15% (95% CI, 14%-17%) had functional iron deficiency. The prevalences of both absolute and functional iron deficiency were higher among women (Figure, A) than men (Figure, B) across all age categories. Among women, the prevalence of absolute iron deficiency was highest between the ages of 18 and 50 years (34%; 95% CI, 31%-37%), whereas among men, the prevalence of absolute iron deficiency was highest among those older than 65 years (7%; 95% CI, 5%-10%). Functional iron deficiency was more common than absolute iron deficiency in all age and sex categories except women younger than 50 years (Table 2).

**Figure.**
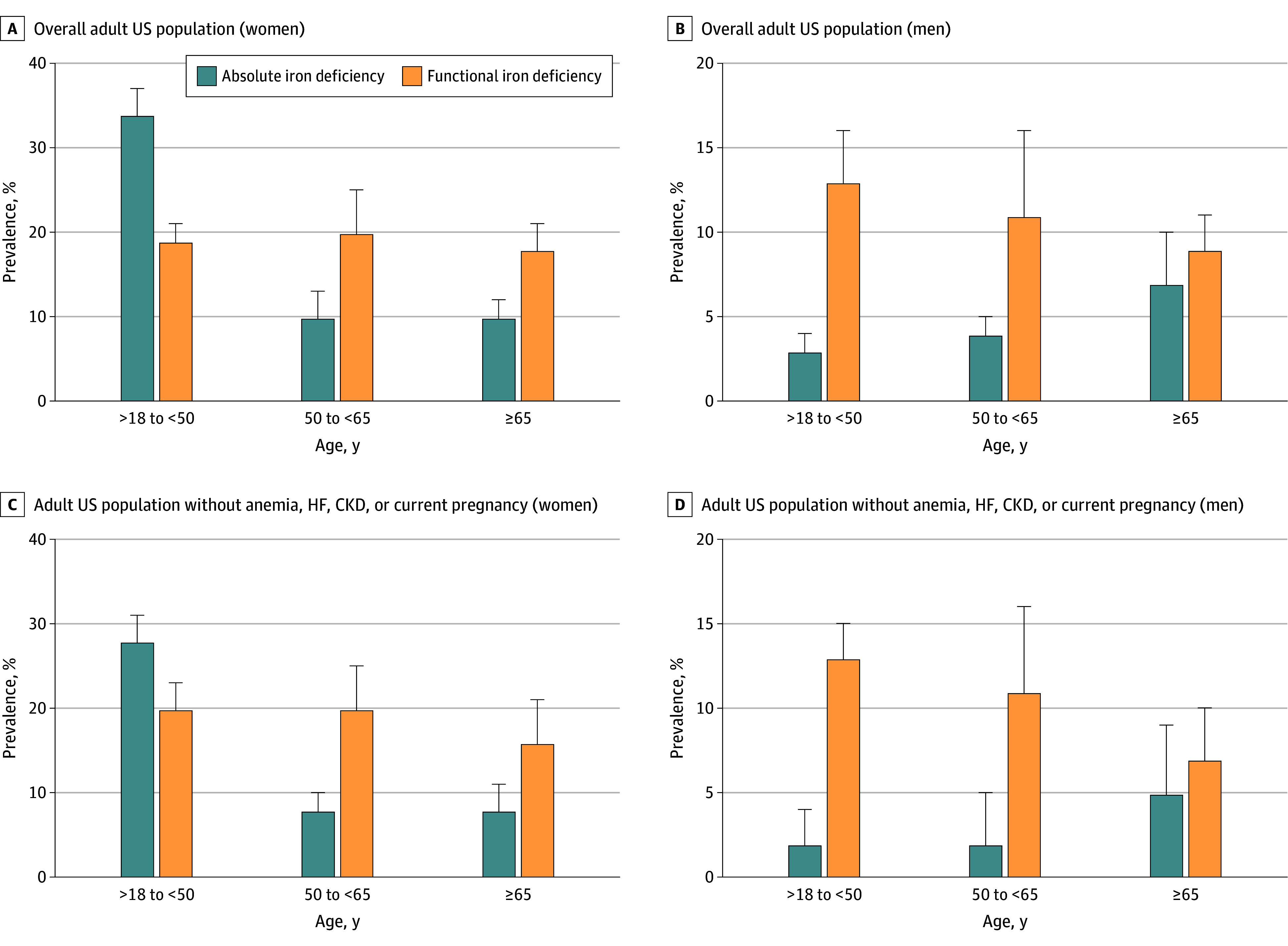
Prevalence of Absolute and Functional Iron Deficiency Among Women and Men Across Age Categories in the US, 2017-2020 Error bars denote 95% CIs. CKD indicates chronic kidney disease; HF, heart failure.

**Table 2.** Estimated Prevalence of Iron Deficiency Among Noninstitutionalized Civilian Women and Men According to Age in the US, 2017-2020

Type of iron deficiency and age category	Participants, % (95% CI)		
	Overall	Women	Men
Absolute iron deficiency			
>18 to <50 y	19 (17-20)	34 (31-37)	3 (2-4)
≥50 to <65 y	7 (6-9)	10 (8-13)	4 (2-5)
≥65 y	8 (7-10)	10 (8-12)	7 (5-10)
Functional iron deficiency			
>18 to <50 y	16 (14-18)	19 (16-21)	13 (11-16)
≥50 to <65 y	16 (13-19)	20 (17-25)	11 (8-16)
≥65 y	14 (12-16)	18 (15-21)	9 (7-11)

In fully adjusted multinomial regression models, the relative risk (RR) of absolute iron deficiency vs iron replete status was significantly higher among women younger than 50 years (RR, 20.79; 95% CI, 10.17-42.48; *P* < .001) and women aged 50 years or older (RR, 2.81; 95% CI, 1.38-5.74; *P* = .006) compared with the reference category of men younger than 50 years of age. Similar results were obtained for the risk of functional iron deficiency vs iron replete status for women younger than 50 years compared with men younger than 50 years (RR, 2.45; 95% CI, 1.54-3.91; *P* = .001) and women aged 50 years or older compared with men younger than 50 years (RR, 1.59; 95% CI, 1.06-2.40; *P* = .03) (Table 3).

**Table 3.** Factors Associated With Absolute and Functional Iron Deficiency vs Iron Replete Status in the US, 2017-2020

Variable	Absolute iron deficiency vs iron replete status		Functional iron deficiency vs iron replete status	
	RR (95% CI)^a^	*P* value	RR (95% CI)^a^	*P* value
Sex and age				
Men aged <50 y	1 [Reference]	NA	1 [Reference]	NA
Men aged ≥50 y	1.00 (0.42-2.38)	.99	0.86 (0.52-1.42)	.55
Women aged <50 y	20.79 (10.17-42.48)	<.001	2.45 (1.54-3.91)	.001
Women aged ≥50 y	2.81 (1.38-5.74)	.006	1.59 (1.06-2.40)	.03
Race and ethnicity				
Asian American	1.29 (0.81-2.05)	.28	1.01 (0.62-1.65)	.95
Mexican American	1.64 (1.16-2.33)	.007	0.92 (0.69-1.24)	.58
Non-Hispanic Black	1.06 (0.82-1.37)	.64	1.13 (0.87-1.47)	.34
Non-Hispanic White	1 [Reference]	NA	1 [Reference]	NA
Other Hispanic	1.37 (0.71-2.65)	.34	1.48 (1.13-1.94)	.007
Any other race or ethnicity not otherwise specified, including multiracial	1.02 (0.47-2.25)	.95	0.80 (0.34-1.42)	.44
Alcohol use				
Nondrinker	1 [Reference]	NA	1 [Reference]	NA
Low-to-moderate	1.55 (0.62-3.89)	.33	0.64 (0.26-1.61)	.33
Heavy	0.72 (0.35-1.44)	.34	0.69 (0.41-1.16)	.15
Body mass index category^b^				
<18.5	0.67 (0.19-2.43)	.53	4.82 (1.12-20.65)	.04
≥18.5 to <24.9	1 [Reference]	NA	1 [Reference]	NA
≥25.0 to <29.9	0.91 (0.61-1.34)	.62	1.60 (1.01-2.56)	.048
≥30.0 to <34.9	0.73 (0.55-0.97)	.03	1.65 (1.07-2.56)	.03
≥34.9 to <40	0.88 (0.52-1.49)	.61	3.13 (1.57-6.24)	.002
≥40	0.54 (0.09-3.11)	.48	2.65 (0.84-8.29)	.09
Anemia, heart failure, chronic kidney disease, or pregnancy				
No	1 [Reference]	NA	1 [Reference]	NA
Yes	4.12 (2.69-6.32)	<.001	1.26 (0.90-1.75)	.17
Dietary iron intake (per doubling)	1.04 (0.81-1.35)	.74	0.86 (0.69-1.08)	.20
Iron supplement use	2.91 (1.73-4.88)	<.001	1.32 (0.73-2.37)	.35
Food security				
High	1 [Reference]	NA	1 [Reference]	NA
Marginal	1.20 (0.76-1.88)	.43	1.42 (0.88-2.29)	.14
Low	1.61 (0.91-2.86)	.10	1.07 (0.61-1.88)	.81
Very low	1.48 (0.70-3.13)	.30	0.92 (0.57-1.50)	.74

Abbreviations: NA, not applicable; RR, relative risk.

^a^ RRs were estimated from multinomial regression model including sex, age, the interaction between sex and age, race and ethnicity, alcohol use, body mass index, presence of 1 comorbidity (anemia, heart failure, chronic kidney disease, or pregnancy), dietary iron intake (per doubling), iron supplement use, and food security status.

^b^ Body mass index is calculated as weight in kilograms divided by height in meters squared.

An estimated 33% (95% CI, 29%-36%) of adults with absolute iron deficiency and 14% (95% CI, 12%-18%) of adults with functional iron deficiency had a potential indication for iron deficiency screening, including prevalent anemia, heart failure, chronic kidney disease, or current pregnancy (eTable 1 in Supplement 1). Among US adults without anemia, heart failure, chronic kidney disease, or current pregnancy, the estimated prevalence of absolute iron deficiency was 11% (95% CI, 10%-11%) and that of functional iron deficiency was 15% (95% CI, 14%-17%). The age-based and sex-based patterns of absolute and functional iron deficiency in the subgroup without anemia, heart failure, chronic kidney disease, or current pregnancy were consistent with those observed in the overall cohort (Figure, C and D). Overall results were similar when using a serum ferritin threshold of less than 15 ng/mL (eTable 2 in Supplement 1).

### Factors Associated With Iron Deficiency Status

eTable 3 in Supplement 1 summarizes participant characteristics according to iron deficiency status. Higher body mass index was associated with a higher risk of functional iron deficiency compared with iron replete status (Table 3) and with a lower transferrin saturation (eTable 4 in Supplement 1). Higher body mass index was associated with higher serum ferritin levels (eTable 4 in Supplement 1). Individuals with anemia, heart failure, chronic kidney disease, or current pregnancy were more likely to have absolute, but not functional, iron deficiency than those without such conditions (Table 3). Dietary iron intake, alcohol use, and food security were not associated with absolute iron deficiency or functional iron deficiency (Table 3).

### Iron Supplement Use and Dietary Iron Intake According to Iron Deficiency Status

eTable 5 in Supplement 1 summarizes the rates of iron supplement use among women and men with and without iron deficiency. Iron supplement use ranged from 22% (95% CI, 12%-37%) to 35% (95% CI, 29%-42%) among women with iron deficiency and from 12% (95% CI, 5%-21%) to 18% (95% CI, 8%-32%) among men with iron deficiency, depending on age. In fully adjusted models, absolute iron deficiency was associated with a nearly 3-fold higher rate of iron supplement use than iron replete status (RR, 2.91; 95% CI, 1.74-4.87; *P* < .001) (Table 4). There was no association between age and sex category with iron supplement use (Table 4). Estimated dietary iron intake was significantly lower among women younger than 50 years or 50 years and older compared with men younger than 50 years in fully adjusted models (Table 4). Marginal food security, but not low or very low food security, was significantly associated with lower dietary iron intake (Table 4).

**Table 4.** Factors Associated With Iron Supplement Use and Dietary Iron Intake in the US, 2017-2020

Variable	Iron supplement use		Dietary iron intake	
	OR (95% CI)	*P* value	β (95% CI)^a^	*P* value
Sex and age				
Men aged <50 y	1 [Reference]	NA	0 [Reference]	NA
Men aged ≥50 y	1.06 (0.46 to 2.43)	.89	−0.05 (−0.12 to 0.03)	.20
Women aged <50 y	1.50 (0.74 to 3.06)	.25	−0.41 (−0.52 to −0.31)	<.001
Women ≥50 y	0.91 (0.48 to 1.72)	.76	−0.40 (−0.47 to −0.34)	<.001
Race and ethnicity				
Asian American	0.49 (0.19 to 1.28)	.14	0.04 (−0.06 to 0.14)	.39
Mexican American	1.38 (0.79 to 2.43)	.25	0.07 (0.01 to 0.14)	.03
Non-Hispanic Black	1.15 (0.80 to 1.66)	.44	−0.12 (−0.21 to −0.02)	.02
Non-Hispanic White	1 [Reference]	NA	0 [Reference]	NA
Other Hispanic	0.99 (0.59 to 1.64)	.95	−0.02 (−0.13 to 0.08)	.67
Any other race or ethnicity not otherwise specified, including multiracial	0.44 (0.23 to 0.83)	.01	−0.18 (−0.32 to −0.04)	.02
Alcohol use				
Nondrinker	1 [Reference]	NA	0 [Reference]	NA
Low-to-moderate	0.72 (0.29 to 1.75)	.45	0.00 (−0.13 to 0.14)	.96
Heavy	0.66 (0.37 to 1.19)	.16	−0.04 (−0.17 to 0.10)	.57
Body mass index	0.99 (0.97 to 1.02)	.86	0.00 (−0.00 to 0.00)	.43
Anemia, heart failure, chronic kidney disease, or pregnancy				
No	1 [Reference]	NA	0 [Reference]	NA
Yes	2.38 (1.57 to 3.59)	<.001	−0.04 (−0.13 to 0.04)	.30
Dietary iron intake (per doubling)	1.11 (0.86 to 1.42)	.41	NA	NA
Iron supplement use	NA	NA	0.04 (−0.05 to 0.14)	.38
Food security				
High	1 [Reference]	NA	0 [Reference]	NA
Marginal	1.05 (0.58 to 1.93)	.86	−0.09 (−0.17 to −0.01)	.03
Low	0.77 (0.41 to 1.43)	.39	−0.02 (−0.13 to 0.09)	.75
Very low	0.86 (0.49 to 1.49)	.57	−0.07 (−0.29 to 0.15)	.52
Iron deficiency status				
Iron replete	1 [Reference]	NA	0 [Reference]	NA
Absolute iron deficiency	2.91 (1.74 to 4.87)	<.001	0.02 (−0.08 to 0.12)	.70
Functional iron deficiency	1.32 (0.73 to 2.36)	.34	−0.05 (−0.14 to 0.04)	.22

Abbreviations: NA, not applicable; OR, odds ratio.

^a^ β coefficient represents a change in dietary iron intake on the log_2_ scale for a 1-unit change in the respective variable.

## Discussion

Iron deficiency can lead to several adverse health outcomes. Widely available blood biomarkers and dietary interventions, nonprescription iron supplements, and, if necessary, prescription oral and intravenous iron products are available to manage iron deficiency. Prior studies^20^ have shown that iron deficiency affects a large proportion of children and younger women (especially in low-income regions), but the prevalence of iron deficiency, especially functional iron deficiency, outside these groups remains unknown in the US. This cross-sectional study indicates that both absolute and functional iron deficiency affect a large proportion of adults in the US, especially among those without conditions often screened for iron deficiency. Moreover, iron supplement use was infrequent among adults with iron deficiency. Absolute and functional iron deficiency may be a widespread, underrecognized public health problem.

Prevalence studies^20,21^ of absolute iron deficiency in the US have focused primarily on populations who are at high risk of developing iron deficiency or anemia, such as infants, pregnant women, and individuals of childbearing age. We, therefore, examined the prevalence of both absolute and functional iron deficiency among women and men older than 18 years with and without known risk factors for iron deficiency.^22^ We found that absolute and functional iron deficiency affect 14% and 15%, respectively, of the US adult general population, even those without risk factors. Our findings agree with results from studies^23,24^ of generally healthy populations in Europe, which also reported high rates of absolute iron deficiency. Together, these studies indicate that the general population has considerable risk for absolute and functional iron deficiency.

The Centers for Disease Control and Prevention guidelines^25,26^ on the screening and prevention of iron deficiency provide recommendations for high-risk groups, but lack clear recommendations for iron deficiency surveillance in the general population. Current screening recommendations^27^ may miss 70% of iron deficiency cases among children and during pregnancy. Our study extends those findings by demonstrating high rates of absolute and functional iron deficiency among adult women and men in the general population, even those without anemia, heart failure, or chronic kidney disease. Studies to determine the optimal absolute iron deficiency screening strategy among adults in the US are needed.

Absolute iron deficiency can be masked in inflammatory states because ferritin acts as an acute phase reactant. In our study, lower mean red blood cell volumes, mean red blood cell hemoglobin levels, and red blood cell distribution widths suggest that some individuals within the functional iron deficiency category had concomitant absolute iron deficiency. The management of absolute and functional iron deficiency differs in important ways. The primary treatment of absolute iron deficiency is iron repletion. In contrast, functional iron deficiency often results from an inflammatory comorbidity. Thus, the management of functional iron deficiency requires identification of the underlying cause (and treatment, if possible), as well as determining the presence of concomitant absolute iron deficiency.

We hypothesized that dietary iron intake, food security, and alcohol use would be associated with iron deficiency but did not find clear patterns to support these nutritional factors as potential causes. A prior study^28^ of pregnant women in the US found an association between food insecurity and iron deficiency. Differences in the populations under study and frequencies of absolute iron deficiency may explain the divergent results in our analysis of food insecurity. Another study^29^ found that increases in the prevalence of iron deficiency anemia paralleled decreases in dietary iron content. Instead, we found that a higher body mass index was associated with a higher risk for functional iron deficiency and lower transferrin saturation. Obesity-related inflammation increases hepcidin expression, and diet-induced weight loss improves iron status in patients with obesity and iron deficiency anemia.^30,31^ Higher body mass index may explain the high prevalence of functional iron deficiency, especially in younger adults without other inflammatory comorbidities. This relationship warrants further study given the increasing prevalence of obesity among adults in the US and the association between functional iron deficiency and adverse health outcomes.^1,32,33^ Among older women and men, comorbidities of aging may be associated with functional iron deficiency, whereas occult gastrointestinal bleeding and poor dietary intake and absorption may be responsible for absolute iron deficiency. Alternative causes of iron deficiency, such as occult gastrointestinal blood loss and intense exercise, were not assessed.

Our study also identified age-related and sex-related patterns in the prevalence of absolute and functional iron deficiency. The prevalence of absolute iron deficiency was highest among women younger than 50 years, possibly due to heavy menstrual bleeding, as suggested by a prior study^20^ of female individuals between the ages of 12 and 21 years. Among older adults, absolute iron deficiency may be related to occult bleeding, whereas functional iron deficiency may be related to comorbidities that increase hepcidin levels. Since the causes, and potentially the treatments, for absolute and functional iron deficiency differ, these age-related and sex-related patterns may have implications for the management of iron deficiency.

### Limitations

Our study has certain limitations. The exact causes of iron deficiency were not systematically ascertained in NHANES. Repeated measures of ferritin and transferrin saturation levels were not available, so trends over time were not feasible. These results are based on a 3-year period of data. The number of people sampled from certain subgroups was small and led to imprecise estimates. We assessed dietary iron intake, but did not account for factors that may influence dietary iron absorption, such as dietary vitamin C content.

## Conclusions

In conclusion, absolute and functional iron deficiency are common among adult women and men in the US, even in the absence of anemia, heart failure, chronic kidney disease, or current pregnancy. Further research on the role of functional iron deficiency in adverse health outcomes and on iron deficiency screening strategies is needed.

## Notes

Supplement 1. **eFigure**. Study cohort selection **eTable 1.** Prevalence of anemia, heart failure and estimated glomerular filtration rate according to iron deficiency status in the United States 2017-2020 **eTable 2.** Estimated prevalence of iron deficiency among non-institutionalized civilian women and men according to age in the United States 2017-2020 **eTable 3.** Characteristics of included adult participants in the NHANES 2017-2020 prepandemic cycle according to iron status **eTable 4.** Participant characteristics associated with log2-serum ferritin and transferrin saturation levels in the United States 2017-2020 **eTable 5.** Iron supplement usage among women and men with and without iron deficiency in the United States in 2017-2020

Supplement 2.
Data Sharing Statement
