# Error in Methods

**DOI:** 10.1001/jamanetworkopen.2024.45802

**Published:** 2024-10-28

In the Original Investigation titled “Absolute and Functional Iron Deficiency in the US, 2017-2020,”^1^ published September 24, 2024, there was an error in the definition of functional iron deficiency in the Methods. The threshold should have been transferrin saturation less than 20%, not less than 30%. This article has been corrected.^1^
