# Supplementary material for: Absolute and Functional Iron Deficiency in the US, 2017-2020
Source: JAMA Netw Open. 2024 Sep 24;7(9):e2433126. doi: 10.1001/jamanetworkopen.2024.33126 (PMC11423176; doi:10.1001/jamanetworkopen.2024.33126)
Supplement: Supplement 1. — eFigure. Study cohort selection eTable 1. Prevalence of anemia, heart failure and estimated glomerular filtration rate according to iron deficiency status in the United States 2017-2020 eTable 2. Estimated prevalence of iron deficiency among non-institutionalized civilian women and men according to age in the United States 2017-2020 eTable 3. Characteristics of included adult participants in the NHANES 2017-2020 prepandemic cycle according to iron status eTable 4. Participant characteristics associated with log2-serum ferritin and transferrin saturation levels in the United States 2017-2020 eTable 5. Iron supplement usage among women and men with and without iron deficiency in the United States in 2017-2020 [file jamanetwopen-e2433126-s001.pdf]

Supplemental Online Content

Tawfik YMK, Billingsley Hayley, Bhatt AS, et al. Absolute and functional iron deficiency in the US, 2017-2020. *JAMA Netw Open*. 2024;7(9):e2433126.
doi:10.1001/jamanetworkopen.2024.33126

eFigure. Study cohort selection
eTable 1. Prevalence of anemia, heart failure and estimated glomerular filtration rate according to iron deficiency status in the United States 2017-2020
eTable 2. Estimated prevalence of iron deficiency among non-institutionalized civilian women and men according to age in the United States 2017-2020
eTable 3. Characteristics of included adult participants in the NHANES 2017-2020 prepandemic cycle according to iron status
eTable 4. Participant characteristics associated with log2-serum ferritin and transferrin saturation levels in the United States 2017-2020
eTable 5. Iron supplement usage among women and men with and without iron deficiency in the United States in 2017-2020

This supplemental material has been provided by the authors to give readers additional information about their work.

© 2024 Tawfik YMK et al. *JAMA Network Open.*

eFigure. Study cohort selection

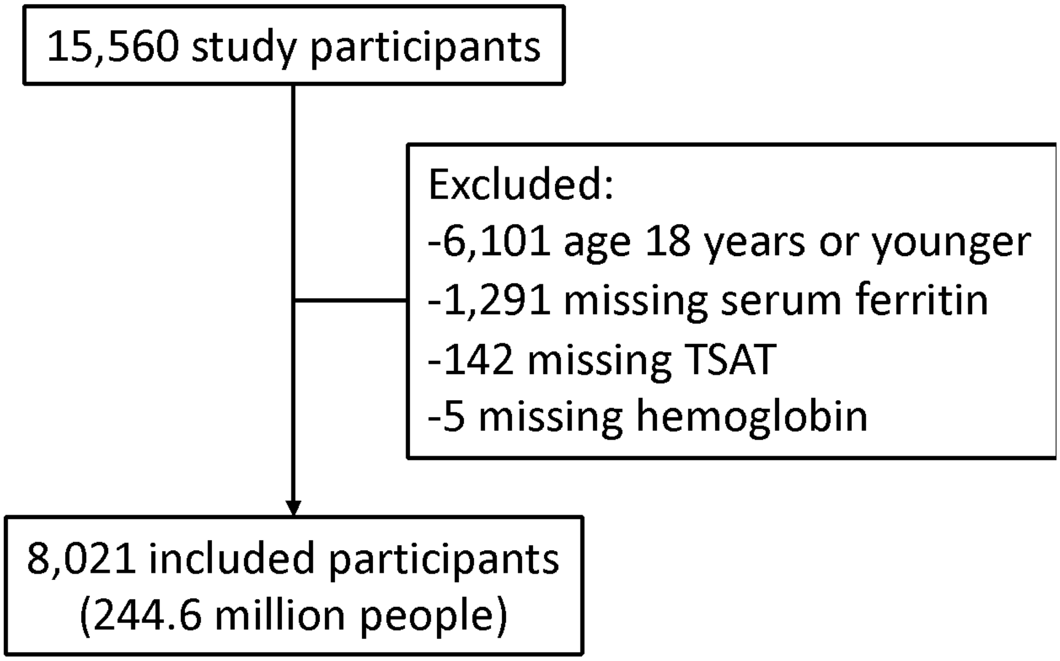

© 2024 Tawfik YMK et al. *JAMA Network Open.*

eTable 1. Prevalence of anemia, heart failure and estimated glomerular filtration rate according to iron deficiency status in the United States 2017-2020

*confidence intervals do not meet National Center for Health Statistics Data Presentation Standards and should be interpreted cautiously

eGFR = estimated glomerular filtration rate

	**Women**			**Men**		
**Age Category**	**Iron Replete**	**Absolute Iron Deficiency**	**Functional Iron Deficiency**	**Iron Replete**	**Absolute Iron Deficiency**	**Functional Iron Deficiency**
Anemia						
> 18 to < 50 years	2% (1%-3%)	26% (20%-32%)	4% (2%-6%)	4% (3%-6%)	24% (11%-41%)	5% (3%-10%)
≥ 50 to < 65 years	3% (2%-4%)	29% (16%-46%)	7% (4%-11%)	7% (5%-9%)	54% (25%-81%)*	17% (10%-27%)
≥ 65 years	7% (4%-10%)	20% (11%-32%	12% (6%-19%)	19% (16%-22%)	47% (26%-69%)*	31% (20%-44%)
Heart Failure						
> 18 to < 50 years	0% (0%-1%)	0% (0%-1%)	1% (0%-3%)	1% (0%-1%)	0% (0%-9%)*	1% (0%-4%)
≥ 50 to < 65 years	1% (0%-3%)	1% (1%-6%)	4% (2%-7%)	5% (2%-8%)	4% (0%-17%)*	9% (3%-19%)*
≥ 65 years	6% (4%-8%)	9% (4%-19%)	9% (5%-15%)	6% (5%-9%)	7% (2%-16%)*	17% (7%-32%)
eGFR less than 60 mL/min per 1.73 m^2						
> 18 to < 50 years	0% (0%-1%)	1% (0%-2%)	1% (0%-2%)	1% (0%-1%)	0% (0%-9%)*	1% (0%-3%)
≥ 50 to < 65 years	3% (2%-5%)	3% (0%-10%)	5% (3%-9%)	3% (2%-5%)	3% (0%-15%)*	4% (1%-8%)*
≥ 65 years	23% (20%-27%)	25% (15%-38%)	28% (22%-35%)	20% (17%-23%)	16% (6%-31%)*	19% (11%-29%)

© 2024 Tawfik YMK et al. *JAMA Network Open.*

eTable 2. Estimated prevalence of iron deficiency among non-institutionalized civilian women and men according to age, sex and comorbidity status in the United States 2017-2020

CI = confidence interval

	Women			Men		
	Overall	Without anemia, HF, CKD and pregnancy	With anemia, HF, CKD or pregnancy	Overall	Without anemia, HF, CKD and pregnancy	With anemia, HF, CKD or pregnancy
**Age Category**	**Percentage (95% CI)**	**Percentage (95% CI)**	**Percentage (95% CI)**	**Percentage (95% CI)**	**Percentage (95% CI)**	**Percentage (95% CI)**
**Absolute Iron Deficiency (Ferritin < 30 ng/mL)**						
> 18 to < 50 years	34 (31-37)	28 (26-31)	80 (73-85)	3 (2-4)	2 (1-4)	14 (6-26)
≥ 50 to < 65 years	10 (8-13)	8 (6-10)	32 (21-45)	4 (2-5)	2 (1-6)	12 (3-30)
≥ 65 years	10 (8-12)	8 (6-11)	13 (9-17)	7 (5-10)	6 (4-10)	9 (5-15)
**Functional Iron Deficiency (Ferritin < 30 ng/mL)**						
> 18 to < 50 years	19 (16-21)	20 (17-23)	9 (6-15)	13 (11-16)	13 (10-15)	14 (6-26)
≥ 50 to < 65 years	20 (17-25)	20 (16-25)	25 (19-32)	11 (8-16)	11 (7-16)	14 (8-24)
≥ 65 years	18 (15-21)	16 (12-21)	22 (17-27)	9 (7-11)	8 (5-10)	12 (8-16)
**Absolute Iron Deficiency (Ferritin < 15 ng/mL)**						
> 18 to < 50 years	14 (12-17)	8 (7-10)	60 (51-67)	1 (0-2)	1 (0-2)	11 (4-25)
≥ 50 to < 65 years	4 (3-6)	2 (1-5)	25 (14-38)	1 (0-2)	0 (0-1)	6 (2-12)
≥ 65 years	2 (1-3)	1 (0-3)	3 (1-5)	1 (0-2)	0 (0-1)	3 (1-7)
**Functional Iron Deficiency (Ferritin < 15 ng/mL)**						
> 18 to < 50 years	30 (27-32)	30 (27-34)	26 (20-32)	13 (11-16)	13 (11-16)	14 (6-26)
≥ 50 to < 65 years	23 (19-27)	22 (18-26)	29 (22-36)	14 (10-18)	13 (9-18)	21 (9-37)
≥ 65 years	22 (19-26)	20 (15-24)	29 (24-34)	12 (9-15)	10 (7-13)	17 (12-22)

© 2024 Tawfik YMK et al. *JAMA Network Open.*

eTable 3. Characteristics of included adult participants in the NHANES 2017-2020 prepandemic cycle according to iron status

FPL = federal poverty level; ASCVD = atherosclerotic cardiovascular disease; CI = confidence interval; DBP = diastolic blood pressure; eGFR = estimated glomerular filtration rate; DFE = dietary folate equivalent; N/A = not applicable; SBP = systolic blood pressure

**Characteristic**	**Normal Iron Status**				**Absolute Iron Deficiency**				**Functional Iron Deficiency**			
	**Men**		**Women**		**Men**		**Women**		**Men**		**Women**	
	**Age >18 to < 50**	**Age ≥ 50**	**Age >18 to < 50**	**Age ≥ 50**	**Age >18 to < 50**	**Age ≥ 50**	**Age >18 to < 50**	**Age ≥ 50**	**Age >18 to < 50**	**Age ≥ 50**	**Age >18 to < 50**	**Age ≥ 50**
Race/Ethnicity, n (%)												
Mexican American	13 (9-18)	5 (4-7)	10 (7-14)	4 (3-6)	20 (5-44)*	1 (0-6)*	14 (9-19)	6 (3-11)	8 (3-14)*	6 (3-11)	11 (7-16)	6 (4-8)
Other Hispanic	9 (7-12)	6 (4-8)	7 (5-10)	6 (5-8)	8 (1-26)*	5 (2-11)*	12 (8-16)	7 (4-11)	10 5-17)	10 (4-16)*	7 (5-11)	7 (5-10)
Non-Hispanic White	57 (51-62)	72 (66-77)	61 (55-66)	72 (66-77)	60 (37-80)*	78 (67-87)	49 (40-58)	72 (65-78)	51 (47-69)	57 (43-70)	59 (52-66)	63 (56-70)
Non-Hispanic Black	11 (8-14)	8 (6-11)	10 (7-13)	10 (7-14)	7 (1-20)*	6 (2-13)*	16 (12-20)	8 (5-13)	12 (7-18)	14 (9-20)	13 (9-18)	14 (10-20)
Non-Hispanic Asian	6 (4-8)	5 (3-7)	6 (4-9)	5 (4-8)	6 (1-18)*	3 (1-9)*	7 (5-10)	5 (3-10)	6 (3-10)	5 (2-8)	5 (3-9)	6 (4-9)
Other, including multi-racial	4 (3-5)	4 (3-6)	5 (4-7)	3 (2-4)	0 (0-9)*	6 (1-20)*	3 (2-4)	2 (0-6)*	6 (3-12)*	9 (3-19)*	5 (2-9)*	4 (2-8)*
Food Security Status, n (%)												
Full Food Security	70 (64-74)	82 (78-86)	70 (63-75)	80 (75-83)	54 (23-82)*	81 (70-89)	59 (52-66)	73 (65-80)	65 (53-76)	71 (63-79)	63 (55-70)	73 (66-80)
Marginal Food Security	13 (10-17)	7 (4-10)	11 (8-15)	9 (6-12)	8 (1-25)*	8 (3-16)*	15 (12-19)	7 (3-14)*	14 (7-24)	11 (7-18)	17 (11-23)	10 (7-14)
Low Food Security	9 (7-11)	5 (4-7)	10 (7-130	7 (5-9)	29 (8-60)*	6 (2-15)*	15 (11-21)	12 (7-19)	12 (7-20)	9 (5-14)	10 (6-14)	8 (5-13)
Very Low Food Security	8 (7-10)	6 (4-8)	9 (6-14)	5 (2-8)	10 (0-46)*	5 (1-13)*	10 (6-15)	8 (3-18)*	9 (4-16)*	8 (3-19)*	11 (7-15)	9 (5-13)
Family Income:Poverty Ratio, n (%)												
≤130%	20 (17-24)	13 (10-17)	21 (17-26)	15 (12-18)	21 (5-48)*	18 (10-28)	29 (24-34)	16 (11-23)	20 (15-27)	15 (10-22)	29 (22-36)	21 (16-27)
130% to 185%	9 (7-11)	9 (7-11)	10 (7-14)	11 (10-13)	4 (0-18)*	8 (2-24)*	12 (9-16)	14 (8-23)	8 (4-13)	13 (8-21)	8 (5-13)	14 (10-18)
186% to 300%	19 (15-22)	19 (15-22)	15 (12-18)	19 (16-23)	7 (1-25)*	21 (12-33)	16 (13-20)	13 (7-23)	14 (9-21)	21 (12-33)	18 (13-25)	19 (14-26)

© 2024 Tawfik YMK et al. *JAMA Network Open.*

>300%	52 (46-57)	59 (55-64)	54 (47-61)	55 (50-60)	68 (41-88)*	53 (33-72)*	43 (35-51)	56 (45-67)	58 (49-66)	51 (39-62)	45 (37-53)	46 (38-54)
Alcohol use, n (%)												
None	5 (3-7)	4 (2-5)	6 (4-8)	10 (8-12)	5 (1-17)*	8 (2-20)*	8 (6-11)	9 (3-17)*	9 (5-16)	10 (2-26)*	7 (4-10)	13 (9-17)
Low-Moderate	42 (36-48)	50 (44-56)	29 (23-36)	36 (32-39)	59 (41-75)*	55 (40-69)	28 (24-33)	29 (22-38)	35 (25-46)	34 (23-48)	25 (19-31)	34 (28-39)
Heavy	53 (48-59(	47 (41-52)	65 (57-72)	55 (51-58)	36 (21-54)*	37 (24-53)	64 (58-69)	62 (52-71)	56 (48-63)	56 (48-64)	69 (62-75)	53 (47-60)
Current cigarette smoking, n (%)	21 (17-26)	14 (11-18)	20 (15-25)	12 (10-15)	22 (7-45)*	13 (6-25)*	15 (11-21)	12 (5-21)*	23 (13-37)	18 (12-26)	14 (7-24)	12 (9-17)
ASCVD, n (%)	1 (1-2)	18 (15-22)	1 (1-2)	12 (9-17)	0 (0-9)*	36 (18-58)*	1 (0-2)	14 (7-25)*	2 (1-5)	20 (11-32)	3 (1-5)	15 (11-19)
Hypertension, n (%)	42 (37-47)	70 (65-74)	31 (26-36)	72 (68-75)	46 (23-70)*	82 (69-91)	29 (24-33)	72 (62-80)	52 (44-60)	81 (72-88)	38 (30-46)	80 (74-86)
Heart failure, n (%)	1 (0-1)	5 (4-7)	0 (0-1)	3 (2-5)	0 (0-9)*	6 (2-12)*	0 (0-1)	5 (2-9)	1 (0-4)	12 (6-20)	1 (0-3)	6 (4-9)
Type 2 Diabetes Mellitus, n (%)	3 (2-5)	23 (21-25)	1 (1-2)	15 (13-18)	0 (0-9)*	52 (30-72)*	3 (2-5)	14 (9-20)	5 (2-10)*	26 (16-38)	6 (3-12)*	27 (21-33)
Body mass index, kg/m^2	29 (0)	29 (0)	29 (0)	30 (0)	30 (1)	30 (1)	30 (1)	29 (1)	32 (1)	31 (1)	33 (1)	32 (1)
eGFR, mL/min per 1.73 m^2	107 (1)	83 (0)	109 (1)	83 (1)	106 (4)	84 (3)	110 (1)	86 (1)	107 (1)	84 (2)	110 (1)	82 (1)
eGFR < 60 mL/min per 1.73 m^2, n (%)	1 (0-1)	10 (8-12)	0 (0-1)	13 (11-15)	0 (0-9)*	10 (5-20)*	1 (0-2)	13 (8-19)	1 (0-3)	9 (6-14)	1 (0-2)	15 (11-19)
hsCRP, mg/L	2.3 (0.1)	2.9 (0.2)	3.3 (0.2)	3.2 (0.2)	1.7 (0.3)	3.3 (0.5)	3.0 (0.3)	3.1 (0.5)	5.8 (0.7)	7.8 (1.2)	7.4 (0.5)	8.3 (1.4)
Serum ferritin, ng/L	204 (7)	230 (6)	86 (4)	144 (5)	23 (2)	21 (1)	19 (0.3)	20 (1)	160 (11)	165 (19)	72 (3)	111 (6)
Transferrin saturation, %	33.9 (0.5)	33.2 (0.5)	32.0 (0.6)	30.0 (0.3)	25.0 (4.6)	19.6 (1.7)	20.1 (0.6)	21.5 (1.3)	16.5 (0.2)	16.2 (0.3)	14.9 (0.2)	16.0 (0.2)
Transferrin receptor, mg/L	--	--	2.57 (0.03)	--	--	--	3.54 (0.06)	--	--	--	3.22 (0.06)	--
Red blood cells, 10^6 cells/mcL	5.08 (0.01)	4.94 (0.02)	4.54 (0.02)	4.58 (0.01)	5.17 (0.16)	4.86 (0.07)	4.51 (0.02)	4.58 (0.06)	5.09 (0.04)	4.96 (0.04)	4.62 (0.02)	4.61 (0.03)
Hemoglobin, g/dL	15.31 (0.04)	15.20 (0.05)	13.81 (0.04)	13.93 (0.05)	14.63 (0.14)	14.18 (0.13)	13.17 (0.06)	13.42 (0.13)	14.89 (0.10)	14.85 (0.12)	13.60 (0.08)	13.46 (0.05)
Hematocrit, %	44.93 (0.13)	44.64 (0.17)	40.81 (0.11)	41.35 (0.14)	43.43 (0.69)	42.54 (0.43)	39.36 (0.16)	40.23 (0.38)	44.08 (0.35)	43.87 (0.34)	40.41 (0.20)	40.35 (0.16)
Mean cell volume, fL	88.70 (0.22)	90.61 (0.21)	90.08 (0.21)	90.61 (0.30)	84.46 (1.46)	87.93 (0.93)	87.56 (0.34)	88.18 (0.81)	86.80 (0.42)	88.62 (0.33)	87.73 (0.45)	87.86 (0.33)
Mean cell hemoglobin concentration, g/dL	34.08 (0.07)	34.03 (0.07)	33.84 (0.06)	33.69 (0.07)	33.69 (0.07)	33.76 (0.51)	33.32 (0.14)	33.36 (0.08)	33.35 (0.12)	33.79 (0.08)	33.64 (0.10)	33.34 (0.08)

© 2024 Tawfik YMK et al. *JAMA Network Open.*

Mean cell hemoglobin, pg	30.32 (0.09)	30.84 (0.08)	30.48 (0.08)	30.52 (0.12)	28.55 (0.88)	29.30 (0.34)	29.30 (0.10)	29.43 (0.34)	29.34 (0.17)	30.01 (0.16)	29.52 (0.20)	29.30 (0.14)
Red cell distribution width, %	13.32 (0.02)	13.55 (0.03)	13.14 (0.03)	13.52 (0.04)	14.42 (0.3)	14.93 (0.40)	14.02 (0.08)	14.65 (0.27)	13.64 (0.09)	13.86 (0.14)	13.52 (0.05)	14.02 (0.07)

© 2024 Tawfik YMK et al. *JAMA Network Open.*

eTable 4. Participant characteristics associated with log2-serum ferritin and transferrin saturation levels in the United States 2017-2020

CI = confidence interval; HF = heart failure; CKD = chronic kidney disease

* beta coefficient represents a change in serum ferritin on the log_2 scale for a 1-unit change in the respective variable

^ beta coefficient represents a 1% change in transferrin saturation for a 1-unit change in the respective variable

	**Log_2-Serum Ferritin**		**Transferrin Saturation**	
**Variable**	**Beta Coefficient* (95% CI)**	**P-Value**	**Beta Coefficient^ (95% CI)**	**P-Value**
**Sex and Age**				
Men younger than 50 years	Reference	---	Reference	---
Men 50 years or older	0.06 (-0.15 to 0.27)	0.56	-0.0 (-2.4 to 2.4)	0.99
Women younger than 50 years	-1.75 (-1.89 to -1.60)	<0.001	-5.5 (-7.6 to -3.3)	<0.001
Women 50 years or older	-0.64 (-0.78 to -0.49)	<0.001	-3.3 (-5.3 to -1.4)	0.002
**Race/Ethnicity**				
White	Reference	---	Reference	---
Mexican American	-0.10 (-0.27 to 0.059)	0.20	0.74 (-1.06 to 2.54)	0.41
Other Hispanic	-0.03 (-0.31 to 0.25)	0.83	-1.08 (-2.36 to 0.19)	0.092
Non-Hispanic Black	0.07 (-0.03 to 0.18)	0.17	-0.96 (-2.09 to 0.18)	0.094
Non-Hispanic Asian	0.10 (-0.08 to 0.28)	0.28	-1.27 (-2.91 to 0.37)	0.12
Other	0.15 (-0.13 to 0.43)	0.29	0.14 (-2.40 to 2.68)	0.91
**Alcohol Use**				
Non-drinker	Reference	---	Reference	---
Low-Moderate	0.00 (-0.31 to 0.32)	0.98	1.52 (-1.06 to 4.11)	0.24
Heavy	0.10 (-0.14 to 0.34)	0.40	1.87 (-0.09 to 3.84)	0.061
**Body mass index (kg/m^2) category**				
< 18.5	0.46 (0.08-0.84)	0.020	-1.46 (-8.74 to 5.82)	0.68
≥ 18.5 to < 24.9	Reference		Reference	
≥ 25.0 to < 29.9	0.26 (0.08-0.45)	0.007	-1.69 (-3.45 to 0.08)	0.060
≥ 30.0 to < 34.9	0.33 (0.15-0.50)	0.001	-2.80 (-4.22 to -1.39)	<0.001
≥ 34.9 to < 40	0.31 (0.11-0.52)	0.004	-5.43 (-6.80 to -4.05)	<0.001
≥ 40	0.11 (-0.41 to 0.64)	0.67	-4.09 (-9.80 to 1.63)	0.15
**Anemia, HF, CKD or Pregnancy**				
No	Reference	---	Reference	---
Yes	-0.58 (-0.76 to -0.40)	<0.001	-5.5 (-6.81 to -4.14)	<0.001
**Dietary iron intake (per doubling)**	-0.11 (-0.21 to -0.00)	0.043	-0.62 (-1.45 to 0.22)	0.14
**Iron Supplement Use**	-0.20 (-0.46 to -0.06)	0.12	-0.98 (-3.16 to 1.19)	0.36
**Food Security**				
High Food Security	Reference	--	Reference	--
Marginal Food Security	0.01 (-0.14 to 0.15)	0.92	-1.71 (-3.23 to -0.19)	0.029
Low Food Security	-0.13 (-0.33 to 0.08)	0.15	-0.81 (-2.52 to 0.91)	0.34
Very Low Food Security	-0.17 (-0.36 to 0.03)	0.086	0.39 (-2.37 to 3.16)	0.77

© 2024 Tawfik YMK et al. *JAMA Network Open.*

eTable 5. Iron supplement usage among women and men with and without iron deficiency in the United States in 2017-2020

CI = confidence interval

	**Women**		**Men**	
	**Iron Deficiency**	**No Iron Deficiency**	**Iron Deficiency**	**No Iron Deficiency**
**Iron Supplement Use, % (95% CI)**				
> 18 to < 50 years	35 (29-42)	32 (26-38)	12 (5-21)	17 (13-22)
≥ 50 to < 65 years	22 (12-37)	26 (20-32)	18 (8-32)	19 (15-24)
≥ 65 years	29 (21-40)	26 (20-33)	17 (8-29)	17 (11-25)
**Daily Dietary Iron Intake, mg**				
> 18 to < 50 years	12 (11-12)	12 (11-13)	16 (15-18)	16 (15-17)
≥ 50 to < 65 years	12 (11-13)	12 (11-12)	16 (15-17)	16 (14-17)
≥ 65 years	12 (11-14)	12 (11-13)	15 (12-17)	16 (15-17)

© 2024 Tawfik YMK et al. *JAMA Network Open.*
