# Supplementary material for: Absolute and Functional Iron Deficiency in the US, 2017-2020
Source: JAMA Netw Open. 2024 Sep 24;7(9):e2433126. doi: 10.1001/jamanetworkopen.2024.33126 (PMC11423176; doi:10.1001/jamanetworkopen.2024.33126)
Supplement: Supplement 2. — Data Sharing Statement [file jamanetwopen-e2433126-s002.pdf]

Data Sharing Statement

Tawfik. Absolute and Functional Iron Deficiency in the US, 2017-2020. *JAMA Netw Open*.
Published September 24, 2024. doi:10.1001/jamanetworkopen.2024.33126

Data
Data available: Data can be obtained from the Centers for Disease Control and Prevention website: https://wwwn.cdc.gov/nchs/nhanes/Default.aspx.
